# School readiness among vulnerable children: a systematic review of studies using a person-centered approach

**DOI:** 10.1186/s41155-024-00298-y

**Published:** 2024-04-17

**Authors:** Gabrielle Garon-Carrier, Corinne Mavungu-Blouin, Marie-Josée Letarte, Jasmine Gobeil-Bourdeau, Caroline Fitzpatrick

**Affiliations:** 1 Département de Psychoéducation, Université de Sherbrooke, Université, 2500 Boulevard de l’Université, Sherbrooke, QC J1K 2R1 Canada; 2 Département de L’enseignement Au Préscolaire Et Au Primaire, Université de Sherbrooke, Université, 2500 Boulevard de l’Université, Sherbrooke, QC J1K 2R1 Canada; 3 Department of Childhood Education, University of Johannesburg, Johannesburg, South Africa

**Keywords:** School readiness profiles, Domains of school readiness, Centered-person approach, Academic achievement, Social adjustment

## Abstract

**Background:**

Research has consistently shown that some children are more vulnerable at the time of school readiness. Better understanding the characteristics of these children is therefore important. Most studies have used a variable-based approach, which may mask the presence of small but important subgroups of children with mixed patterns of readiness strengths and weaknesses. Identifying subgroups with mixed readiness patterns using a person-centered approach matters because their developmental trajectories might differ in important ways from children with broader difficulties across all readiness domains.

**Objective:**

This systematic review attempts to synthesize existing profiles of school readiness conducted on preschool-aged children and to describe how these various profiles are associated with children’s academic achievement and social adjustment during their school years. Specifically, we described how the school readiness profiles vary in number of profiles identified and differences in the specific domains of school readiness. We further describe the school readiness profiles and how they predict later academic and social outcomes. Furthermore, we focus on profile differences between at-risk and non-at-risk preschoolers.

**Methods:**

Longitudinal studies published between 2005 and 2022 on profiles of school readiness before school entry and at least one subsequent academic and/or social outcomes were extracted from five databases. Eight articles were included in this systematic review out of the 117 screened peer-reviewed articles.

**Results:**

All the studies incorporated both the cognitive and socioemotional domains of school readiness in their profiles. Fifteen profiles of school readiness at preschool age were identified based on the child level of cognitive and socioemotional skills, with 7 profiles at risk of later academic and social difficulties. Despite variation, children in these at-risk profiles of school readiness shared similar features.

**Conclusion:**

This literature review provides an exhaustive summary on the number of profiles and domains of school readiness most frequently reported in studies using a person-centered approach. Yielding an in-depth description of at-risk profiles of school readiness can help designing early preventive intervention for these children.

**Supplementary Information:**

The online version contains supplementary material available at 10.1186/s41155-024-00298-y.

Child school readiness, defined as their ability to function successfully in the school context, is one key early-life factor enabling well-being, successful psychosocial and school adjustment across the life course (Pan et al., 2023; Rimm-Kaufman & Pianta, 2000; Sabol & Pianta, 2012). Preschool-aged children unprepared for school entry are at risk of lower grades (Fitzpatrick et al., 2020), of school dropout (Fitzpatrick et al., 2020), and of displaying antisocial behaviors (Jones et al., 2015). In contrast, being ready to learn shows several long-term benefits such as greater commitment to learning (Pagani et al., 2010; Pan et al., 2023), higher quality relationships with peers and teachers (Guhn et al., 2016; Sandilos et al., 2019), and better mental and physical health during the transition to adulthood (Fitzpatrick et al., 2020). In the past decade, various profiles of school readiness have been identified among preschool-aged children. This systematic review aims to describe existing profiles of preschoolers’ school readiness and how they associated with subsequent children’s academic and social outcomes, with a particular focus on school readiness profiles most at risk of persistent difficulties.

## What skills make up school readiness?

As a conceptual framework, school readiness has been defined as a holistic and multidimensional concept involving several developmental domains such as cognitive, language, socioemotional, and motor/physical skills (Boivin & Bierman, 2014; Forget-Dubois et al., 2007; Janus & Offord, 2007; Snow, 2006). The cognitive domain refers to ways of thinking and acquiring knowledge that promotes learning. The language domain includes literacy skills that promote effective communication with others. Competence in the cognitive and language domains usually encompasses memory and attentional skills, expressive and receptive vocabulary, and the early numeracy and reading skills, such as knowing numbers and letters (Duncan et al., 2007; Janus & Offord, 2007; Kagan et al., 1995; La Paro & Pianta, 2000; Snow, 2006, 2007). The socioemotional domain refers to the development of key social behaviors that help build and maintain positive relationships with others (Sabol & Pianta, 2012). It usually includes low levels of externalizing (e.g., aggression, opposition) and internalizing (e.g., anxiety, depression) behaviors (Duncan et al., 2007; Janus & Offord, 2007; Kagan et al., 1995; La Paro & Pianta, 2000; Sabol & Pianta, 2012; Snow, 2006). The motor/physical domain refers to children’s health and motor development that support engagement and learning in their environments (Cinar et al., 2023; Pagani et al., 2010). This domain generally involves physical fitness, fine and gross motor skills, and health status.

Although interrelated, each domain is a unique aspect of school readiness. It is now well documented that these domains of school readiness prior school entry are differently associated with later academic achievement and performance at standardized tests (Claessens et al., 2009; Davies et al., 2016; Duncan et al., 2007, 2020; Fitzpatrick et al., 2020; Hamerslag et al., 2018; Pagani et al., 2010), as well as social adjustment, such as the level of social competence and behavior problems (Eisenberg et al., 2010; Robson et al., 2020).

## Children at risk of low school readiness

Children may experience school readiness difficulties, especially during the transition into formal schooling. Teacher reports that 48% of children manifest difficulty adjusting to school during the transition to kindergarten (Rimm-Kaufman et al., 2000), with most frequently reported problems being difficulty following directions or working independently, and a lack of pre-academic skills. For most children, these school readiness difficulties progressively fade away as adaptation unfolds during the elementary school years (Parent et al., 2019; Pingault et al., 2015; Rimm-Kaufman et al., 2000), but some will still experience serious adaptative problems (Simard et al., 2018; Rimm-Kaufman et al., 2000).

In particular, children with expressive or receptive language difficulties, those lagging behind in terms of general knowledge, and children with disruptive behaviors are at higher risk of low school readiness (Abenavoli et al., 2017; Montes et al., 2012). Evidence also suggests that children from minority language (Janus et al., 2010), those from families with low parental education (Montes et al., 2012), low socioeconomic status (Abenavoli et al., 2017; Gullo, 2018; Hartman et al., 2017), and boys encounter heightened risk of poor school readiness (Brandlistuen et al., 2021; Gullo, 2018; Hamerslag et al., 2018; Lewicki et al., 2018).

## Profiles of school readiness

Most studies to date have used a variable-oriented approach which assumes that while individuals differ quantitatively across variables, they are qualitatively alike in the nature of the relationships between variables. As such, a variable-oriented approach may mask the presence of small but important subgroups of children with mixed patterns of school readiness strengths and weaknesses. Identifying subgroups with mixed readiness patterns matters because their developmental trajectories might differ in important ways from children with broader difficulties across all school readiness domains. One way to examine the interplay among school readiness domains is to use a person-centered approach. A person-centered approach confers several advantages over a variable-centered approach (Laursen & Hoff, 2006). This approach allows us to better capture population heterogeneity by identifying subgroups of children that could go undetected in variable/mean-based approaches. Specifically, it allows for the possibility that subgroups of children show distinct trends, thus providing a more nuanced view of children’s level (and development) of school readiness. From a preventive perspective, using a person-centered approach to establish profiles of school readiness among preschoolers may help in identifying children most at-risk of persistent academic and/or social difficulties (Weller et al., 2020). Furthermore, not only is it essential to document the various profiles of school readiness, but it is also crucial to estimate whether and how some of these profiles foresee later academic and social difficulties (Abenavoli et al., 2017).

From a theoretical perspective, a person-centered approach simultaneously considers the multiple cognitive, language, socioemotional, and physical/motor characteristics of the child, which align with the core definition of school readiness as a multidimensional concept (Bergman & Magnusson, 1997; Bergman & Trost, 2006; Laursen & Hoff, 2006; Snow, 2007). From a statistical point of view, this approach does not make strong assumptions about the population distribution of the putative profiles. It also considers individual differences to determine the optimal number of potential profiles by clustering within the same profile preschoolers with similar school readiness attributes (Sandilos et al., 2019).

Using a multidimensional conceptual framework of school readiness, the present article provides a systematic review of studies that have used a person-centered approach to describe profiles of school readiness among preschoolers and their associated later academic and social outcomes. Existing studies on this topic differ from one another on the basis of the dimensions of school readiness being studied and methodological approaches (Gobeil-Bourdeau et al., 2022). For this reason, systematic reviews are important for identifying areas of consensus as well as existing gaps. Specifically, we aim to describe how the profiles identified in the literature vary in the number of profiles identified and differences in the specific dimensions of school readiness. It is expected that each study included in this systematic review will report at least three profiles of school readiness, with a small but significant group of children with a low school readiness profile (Garon-Carrier et al., 2018; Simard et al., 2018). We also qualitatively describe the school readiness profiles and how they predict academic and social outcomes during compulsory education. Furthermore, we focus on profile differences between at-risk and non-at-risk preschoolers. We expected children from linguistically and ethnically diverse families, those with low parental education and socioeconomic status, and a greater proportion of boys than girls to fall on a low/at-risk profile of school readiness.

### Methods

This systematic review follows the propositions for systematic reviews from the University of York (Centre for Reviews and Dissemination, 2009). Systematic reviews adopt a sequential step-by-step rigorous scientific approach to identify and synthesize evidence on a specific research question.

## Step 1: Clarifying the research question

The review aims to describe the various school readiness profiles of preschoolers (i.e., before school entry or prior to age 6) and their associated academic and social outcomes in grade 1 (age 6) or upward. The profiles were obtained with a person-centered statistical approach such as a latent class/profile analysis or a cluster analysis.

## Step 2: Identifying relevant studies

An electronic search was conducted in PsycINFO, ERIC, Academic Search Complete, Education Source, and ProQuest in May 2022. Our search strategies included keywords on school readiness (“school readiness” OR “preschool skills” OR “kindergarten readiness” OR “preschool competenc*”), followed by keywords about the person-centered approach or the type of analysis (“person-centered” OR “person-oriented” OR cluster OR “latent class” or “pattern-based” OR “latent profile”). No keyword about the social and academic outcomes was introduced, as this has been proved too restrictive. However, the studies that we selected (see step 3) included academic achievement and/or social adjustment outcomes. A number of 167 studies came out from this research. Only peer-reviewed articles (*n* = 117) were selected to ensure a minimum of methodological quality.

## Step 3: Selecting relevant studies

Studies had to meet the following criteria: (1) the school readiness profiles had to be the independent variable; (2) in accordance with the multidimensional conceptual definition of school readiness, the profiles had to include at least two domains of school readiness; (3) the profiles had to be conducted before the beginning of the elementary school years; and (4) studies had to examine academic achievement and/or social adjustment from the elementary school years (or upward) as a dependent variable. Studies examining the effectiveness of an intervention program, and studies conducted on children with specific disorders (e.g., autism spectrum disorder, disabilities, traumatic brain injury), were excluded. Only 12 studies were selected based on these criteria. Among the selected studies, two were written by the same authors. These two articles reported school readiness profiles that were conducted on the same sample (sociodemographic similarities). They revealed identical school readiness profiles and had similar associations with later academic achievement. Given the great similarities between these two studies, we kept only one of the two articles, i.e., the one providing the most detailed methodology (Quirk et al., 2013). Three studies out of 11 were also excluded (Goble et al., 2019; Quirk et al., 2016; Sabol & Pianta, 2012) because they reported the same profiles of school readiness that were previously published to examine how these profiles related to different academic or social outcomes. This led to a total of eight selected studies.

## Step 4: Assessing quality of studies

We assessed the quality of the selected studies (*n* = 8) according to their methodological features. Studies needed to include a clear description of the following: (1) sample characteristics (e.g., sex, age, ethnic group, socioeconomic status), (2) indicators of school readiness incorporated in each profiles (e.g., instruments being used), (3) statistical analysis conducted to create the profiles, and (4) measures of academic and/or social outcomes and of the analysis performed to associate the profiles of school readiness with the outcomes. The quality of the selected studies was satisfying and was judge deemed adequate to be included in this systematic review.

## Step 5: Extracting data

We extracted the methodological characteristics of each study. We also highlighted some characteristics of the school readiness profiles identified in each study, such as the number of profiles, the proportion of children in each profile, and their level on each indicator of school readiness. To examine how the school readiness profiles were differentially associated with later academic and/or social outcomes, we extracted the information about the strength and the direction (positive or negative) of these associations.

## Results

### Methodological characteristics of the studies

Table 1 shows the methodological characteristics of the studies (*n* = 8). Most studies reported school readiness profiles conducted on a normative sample (*n* = 5; 62.5%). Three studies created school readiness profiles on at-risk samples (*n* = 3; 37.5%) including either children with language difficulties or from a family with a low socioeconomic status. All studies had a sample with a similar proportion of boys and girls. Between one and seven socioemotional indicators of school readiness were used to create profiles, such as internalizing and externalizing problems (5 out of 8 studies) and social competence (*n* = 4). Most of these indicators were reported by the teacher and the parents. Only one study measured socioemotional indicators of school readiness with direct observation (*n* = 1). Cognitive indicators of school readiness were measured with standardized tests taken by the child (*n* = 6) or reports by the teachers (*n* = 5). Early academic skills, such as knowing numbers, letters, and colors, were reported in six studies, and five studies measured language skills, such as receptive vocabulary, expressive language, and communication skills. Other cognitive indicators of school readiness were also measured in five studies, such as executive functions, problem-solving skills, general knowledge, and IQ.

**Table 1:** Methodological characteristics of the profiles of school readiness

	*N*	**Language skills**		**Cognitive skills**		**Socioemotional skills**	**Motor/physical skills**
				Pre-academic	Other		
Tavassolie et al. (2022)	43,044	ST		ST		P, T	ST
Fitzpatrick (2017)	670	ST		ST	ST	T	
Christensen et al. (2020)	4386	T		T	T	T	T
Hair et al. (2006)	17,219	ST, T		ST, T	ST, T	P, T	ST, P
Quirk et al. (2013)	781	T		T		T	T
Konold and Pianta (2005)	964				ST	P, DO	
McWayne et al. (2012a)	1082			ST	T	P, T	
McWayne et al. (2012b)	2336			ST	T	P, T	

*ST* standardized test administered to the child, *P* data from the parent, *T* data from the teacher, *DO* direct observation

In addition, four studies included the level of children motor/physical skills in their school readiness profiles. One study, unlike others, also conducted school readiness profiles using parental attributes (e.g., parent psychological distress, parenting efficacity) and community characteristics (e.g., neighborhood social capital, quality of school environment).

## Summary of the school readiness profiles

### Differences in the specific dimensions of school readiness

Among the eight selected studies, only half of them included all the domains of school readiness, i.e., the language, cognitive, socioemotional, and motor/physical skills (Christensen et al., 2020; Hair et al., 2006; Quirk et al., 2013; Tavassolie et al., 2022). One study incorporated three domains of school readiness out of four (language, cognitive, socioemotional) in their profiles (Fitzpatrick, 2017). Three studies conducted profiles with only two domains of school readiness, the cognitive and socioemotional domains (Konold & Pianta, 2005; McWayne et al., 2012a, 2012b).

All the studies, however, incorporated both the cognitive and socioemotional domains of school readiness in their profiles, suggesting these two domains as core components of school readiness. For this reason, we next described all the existing profiles from the selected studies based on these two domains of school readiness.

## The number of profiles

Each study reported between 3 and 6 profiles of school readiness. These profiles varied based on the level (high, average, or low) on each domain of school readiness (see Table S1, in supplementary material). Overall, 15 profiles were identified across studies based on the cognitive and socioemotional domains of school readiness. These profiles are shown in Table 2.

**Table 2:** Profiles of school readiness based on the level of cognitive and socioemotional skills

	**Cognitive/low**	**Cognitive/average**	**Cognitive/high**	**Cognitive/mixed**
Socioemotional/low	Low balanced (B-) 1, 2, 3, 4, 2 × 5	Socioemotional difficulty (SE-C) 3, 6	High cognitive and low socioemotional skills (C + SE-) 6	Unbalanced cognition (UC) 7
Socioemotional/average	Cognitive difficulty (C-SE) 4, 6	Moderate balanced (M) 3, 5, 6, 8	Cognitive strength (C + SE) 7	Tend to average (TA) 2, 6
Socioemotional/high	High socioemotional and low cognitive skills (SE + C-) 4, 5	Socioemotional strength (SE + C) 6	High balanced (B +) 2, 3, 4, 5, 8	
Socioemotional/mixed	Tend to difficulties (T-) 2 × 1, 8	Average cognitive with mixed socioemotional skills (SE↕) 2 × 7	Tend to strength (T +) 3 × 1	Unbalanced (U) 7

1, Tavassolie et al. (2022); 2, Fitzpatrick (2017); 3, Christensen et al. (2020); 4, Hair et al. (2006); 5, Quirk et al. (2013); 6, Konold and Pianta (2005); 7, McWayne et al. (2012a); 8, McWayne et al. (2012b)

## Description of school readiness profiles

As shown in Table 2, each profile was characterized by either a high, an average, or a low level on the cognitive and socioemotional domains of school readiness. Profiles of children could also be heterogeneous, which means they had various levels within the cognitive or within the socioemotional aspect of school readiness, depending on the indicators being used. Three profiles of school readiness were qualified as *balanced* since they grouped children with similar levels of cognitive and socioemotional skills (on the diagonal in Table 2). These profiles were the most frequently reported ones (77.7% of studies found at least one balanced profile). For instance, the high-balanced profile (B +) includes children with high levels of cognitive and socioemotional skills. This profile is highly prevalent among studies and has a greater proportion of girls than boys. Four studies also identified a moderate-balanced profile (M) of children with an average level of both cognitive and socioemotional skills. The Low-Balanced profile (B-) includes children with low cognitive and low socioemotional skills. This profile of preschoolers shows the lowest levels of school readiness. It includes between 7 and 31% of children across studies (*n* = 5 out of 8 studies), and as expected, it has a greater proportion of boys than girls. One study had two Low-Balanced profiles that were different based on children’s levels of motor/physical skills (low vs average).

Table 2 also shows six *unbalanced* profiles of school readiness, with different levels of cognitive vs socioemotional skills. Out of them, two profiles were high on one aspect but low on the other. In other words, one profile had high level of socioemotional skills but low level of cognitive abilities (SE + C-), and one profile had high cognitive abilities but low socioemotional skills (C + SE-). Four profiles had an average level on one domain (cognitive or socioemotional) of school readiness and were high (C + SE; SE + C) or low (C-SE; SE-C) on the other domain.

Six *heterogeneous* profiles of school readiness were also reported in studies. For instance, one heterogeneous profile, tend to average (TA), had various levels of skills in the cognitive dimension of school readiness, with children having low receptive vocabulary but an average level of number knowledge (Fitzpatrick, 2017; Konold & Pianta, 2005). The tend to strength profile (T +), found in only one study (Tavassolie et al., 2022), was characterized by having pre-academic strength and strong to average behavior at school or at home. In contrast, the tend to difficulties profile (T-) (McWayne et al., 2012b; Tavassolie et al., 2022) were characterized by low pre-academic skills and low-average socioemotional behavior at school or home. Children in the unbalanced cognition profile (UC) were characterized by low socioemotional skills (i.e., high behavior problems at school as rated by the teacher) with low to average pre-academic skills (McWayne et al., 2012a). The unbalanced profile (U) had high ratings given by teachers for children’s social ability but performed within the average range on independent assessments of emergent literacy and numeracy (McWayne et al., 2012a). The last heterogeneous profile is the average cognitive with mixed socioemotional profile (SE↕) (McWayne et al., 2012a), with children in this profile having a relatively average pre-academic and mixed socioemotional at home (i.e., average social skills but low behavior problems). Is it worth noting that most of these heterogeneous profiles were found in one or two studies, conducted on Head Start children (McWayne et al., 2012a, 2012b), children from low-income, and ethnically and linguistically diverse children (Tavassolie et al., 2022).

## School readiness profiles prior school entry and their associated academic and social outcomes during compulsory education

Among the *balanced* profiles, children in the high-balanced (B +) profile of school readiness prior school entry had the highest levels of academic achievement and social adjustment during compulsory education. In contrast, children in the Low-Balanced (B-) profile of school readiness had the lowest level of later academic achievement and social adjustment. Little difference was found between the moderate-balanced (M) and the high-balanced (B +) profiles of school readiness on their academic and social adjustment during compulsory education.

In the prediction of academic achievement, no significant difference between *unbalanced* profiles of school readiness prior school entry was found when the language component of school readiness was controlled for. Without surprise, children in profiles of school readiness characterized by high socioemotional skills (SE + C-, SE + C) better performed in terms of later social adjustment in comparison to school readiness profiles characterized by lower socioemotional skills (SE-C, UC). The high cognitive with low socioemotional profile (C + SE-) of school readiness was the only exception, suggesting that high cognitive skills during the preschool years could help buffer the lack of socioemotional skills at this age in order to help children’s later social adjustment.

## At-risk profiles of school readiness

Seven profiles of school readiness prior school entry were low on one domain of school readiness (cognitive or socioemotional) or on both domains and thus were considered at risk of later academic and social difficulties. These profiles are described more in-depth. First, the profile of school readiness most at risk of persistent academic and social difficulties during compulsory education was the Low-Balanced profile (B-). Children in this profile had the lowest level of later academic achievement (Christensen et al., 2020; Fitzpatrick, 2017; Hair et al., 2006; Quirk et al., 2013; Tavassolie et al., 2022) and of social skills (Christensen et al., 2020; Hair et al., 2006). This profile usually includes, among a normative population, a small proportion of children (between 7 and 13.24%) with low language skills (Christensen et al., 2020), with low number knowledge and classroom engagement (Fitzpatrick, 2017), and with low scores (2 SD below the mean) on social/emotional development measures (Hair et al., 2006). Children with a Low-Balanced profile of school readiness were, in a greater proportion, non-English language learner (Tavassolie et al., 2022), Latino (Quirk et al., 2013), or Black (Hair et al., 2006; Tavassolie et al., 2022). They were also less likely to have attended a preschool program (Quirk et al., 2013) and had elevated family risk factors such as low socioeconomic status (Christensen et al., 2020; Quirk et al., 2013; Tavassolie et al., 2022), parents with lower educational attainment (Quirk et al., 2013), or parent psychological distress (Christensen et al., 2020). Males and children with a disability were also overrepresented in the Low-Balanced profile of school readiness (Quirk et al., 2013; Tavassolie et al., 2022).

Three profiles of school readiness had low cognitive abilities and thus were at greater risk of struggling at school: a profile with cognitive difficulty (C-SE), one with high socioemotional and low cognitive skills (SE + C-), and one who tend to difficulties (T-). The cognitive difficulty profile (C-SE) of school readiness included between 7.0 and 19.4% of preschoolers (Hair et al., 2006). At preschool age, children in this profile were below the mean on language and cognition (Hair et al., 2006) and had attention problems or low working memory. They were Hispanic or non-White in a greater proportion (Hair et al., 2006). Males, children with a disability, and those from economically disadvantaged households were also overrepresented in this profile (Hair et al., 2006). They also performed poorly at math and reading assessment during the formal school years (Hair et al., 2006). The high socioemotional and low cognitive skills profile (SE + C-) of school readiness was found in only two studies (Hair et al., 2006; Quirk et al., 2013) and included between 17.0 to 33.9% of the preschoolers. These children were, in a greater proportion, from mothers and fathers not speaking English to the child (Hair et al., 2006). They also had significantly lower English proficiency scores prior school entry and less exposure to preschool program (Quirk et al., 2013). However, this group of children did not perform significantly lower than children in the Low-Balanced profile with regard to their later academic achievement (Quirk et al., 2013). Similarly, the tend to difficulties profile (T-) was reported in only two studies (McWayne et al., 2012b; Tavassolie et al., 2022). Children from a linguistic minority as well as ethnical minority such as Latino and Black preschoolers had increased odds of being in this profile of school readiness (McWayne et al., 2012b; Tavassolie et al., 2022). Preschoolers in special education or having a disability (McWayne et al., 2012b; Tavassolie et al., 2022) and those receiving free/reduced lunch (Tavassolie et al., 2022) were also overrepresented in this profile. This profile, although being at risk, had greater academic achievement during compulsory education (grade 3 GPA, reading and math test scores) in comparison to the Low-Balanced profile of school readiness (Tavassolie et al., 2022).

Three additional profiles of school readiness with low socioemotional abilities at preschool age were at greater risk of persistent difficulties during the school years: a profile with socioemotional difficulty (SE-C), one with high cognitive and low socioemotional skills (SE + C-), and one with unbalanced cognition (UC). The socioemotional difficulty profile (SE-C) of school readiness included between 7 and 17% of preschoolers (Christensen et al., 2020; Konold & Pianta, 2005). Children in this profile had an increased likelihood of vulnerability in later social competence and emotional maturity and a poor teacher–child relationship (Christensen et al., 2020). They had the highest proportion of non-White children, and of families classified as poor (Konold & Pianta, 2005), and the lowest home quality score. In terms of academic and social outcomes, this profile of children had the lowest level of engagement at school in Grade 5 (Sabol & Pianta, 2012), but did not differ from other profiles in achievement test performance in Grade 1 (Konold & Pianta, 2005) and in terms of executive functions in Grade 9 (age 15; Goble et al., 2019). The high cognitive and low socioemotional skills profile (SE + C-) of school readiness included about 22% of preschoolers (Konold & Pianta, 2005), with a greater proportion of non-Whites children. They were characterized by elevated reported externalizing behaviors, but they had high working memory (and overall cognitive skills) during the preschool years. Mothers of children in this school readiness profile had more years of education than mothers of children in most of the other school readiness profiles. Interestingly, the SE + C- profile of school readiness had low teacher-reported disruptive behavior disorders during compulsory education, the highest score in math and reading performance in Grade 5 (Sabol & Pianta, 2012), and higher GPA, math and literacy skills, and executive functions in Grade 9 (Goble et al., 2019). In other words, despite having low socioemotional skills at preschool age, children in this profile of school readiness did not manifest later academic or social difficulties. At last, the unbalanced cognition profile (UC), reported in only one study (McWayne et al., 2012a), represented 17% of preschoolers. In this profile, there were significantly more boys, linguistic and ethnic minority children, and children with diagnosed disabilities.

## Discussion

This study advanced knowledge on profiles of school readiness and how it predicts later academic and social adjustment by reviewing studies using a person-centered approach. In this systematic review, we identified 15 profiles of school readiness: 3 balanced, 6 unbalanced, and 6 heterogenous profiles. The balanced profiles were the most frequently reported, while the heterogenous ones were the profiles less frequently reported in studies. Notable differences were found between the 15 profiles of school readiness. By highlighting these findings and by identifying 15 profiles of school readiness, each of them being differently associated with social and academic outcomes, this systematic review supports the relevance of studying school readiness with a person-centered approach. For instance, in the present study, we found evidence that considering profiles of school readiness can help forecast which children will experience difficulties. In particular, we identified seven profiles that were at risk of later academic and social difficulties, with the Low Balanced (B-) being the most at-risk profile. Despite variation in the levels of cognition and/or socioemotional skills, most of these at-risk profiles of school readiness shared similar features. These at-risk profiles of school readiness had subsequent lower academic and/or social outcomes.

These findings can help designing early preventive intervention for children with at-risk profiles of school readiness. Children being in an at-risk profile of school readiness were from a linguistic and/or visible minority family in a greater proportion and had elevated family risk factors such as a low home quality, low socioeconomic status, and low maternal educational attainment. Furthermore, they did not attend a preschool program and/or had a diagnosed disability. Boys also tended to belong to at-risk profiles of school readiness leading to lower academic and social outcomes. Thus, these children should deserve special attention to alleviate later academic and social difficulties. The attention given to these at-risk children should also persist in the early school years.

One potential approach to promote academic and social adjustment among children with initial risk of underachievement is to promote the accessibility to high-quality childcare arrangements. Evidence of preschool childcare attendance on school readiness and social outcomes during childhood has been convincing (Geoffroy et al., 2010; Gomajee et al., 2018; Loeb et al., 2007). Previous studies found direct associations between preschool childcare attendance and children’s later academic and social outcomes (Ansari, 2018; Gomajee et al., 2018; Phillips & Lowenstein, 2011), especially for children of mothers with low levels of education (Geoffroy et al., 2010) and facing economic hardship (Ansari, 2018; Laurin et al., 2015). Parent involvement in intervention program with both home- and school-based components is also another approach to promote school readiness among at-risk children (Marti et al., 2018), as well as programs capitalizing on early childhood educators training (Brown et al., 2009). In particular, programs focusing on responsive parent–child and educator-child interactions as well as parent-educator partnerships have been proved effective (Waters & Catlett, 2020).

## Limitations and recommendations for future studies

This systematic review has limitations that must be mentioned to clearly confine its results. First, only studies undertaking a multidimensional conceptualization of school readiness were selected. These studies had to include at least one cognitive and one socioemotional indicator of school readiness. Similarly, this systematic review only included studies focusing on academic and social outcomes during compulsory education. Thus, it excludes studies investigating other outcomes such as health-related ones and studies focusing on the predictors of school readiness profiles. Second, some specific results from the selected studies, hard to summarize within the current review, were omitted in the tables. We also, sometimes, grouped several profiles obtained in a study under one profile of school readiness. For instance, McWayne et al. (2012a) detected two profiles of school readiness that had similar cognitive levels, but one of these profiles had lower levels of socioemotional skills at home. These two profiles were both grouped in the average cognitive with mixed socioemotional profile. This choice brought a more understandable portrait of the existing literature but also hides some fine nuances found in some studies. Third, this systematic review is mainly based on studies conducted on the general population of children or on children from families with a low socioeconomic status. Other profiles of school readiness, not identified in the current review, might exist among children with specific vulnerabilities. Future literature reviews would benefit from replicating our findings with studies on at-risk children, as different profiles of school readiness could emerge in such populations.

## Conclusion

Examining profiles of school readiness before school entry matters for children’s academic and social adjustment. This systematic review disentangled 15 profiles of school readiness based on the child level of cognitive and socioemotional skills, with 7 profiles being systematically associated with worse academic achievement and social adjustment. Most of these at-risk profiles of school readiness were from a linguistic and/or visible minority family and had elevated family risk factors, and these at-risk children (boys in a greater proportion) did not participate to a preschool program. These findings have implications for early identification of at-risk children, as it gives a clear portrait of children from preschool age showing lower school readiness and higher risk for social and academic underachievement.

This systematic review also highlighted areas of consensus and existing gaps. While all studies incorporated the cognitive and socioemotional dimensions of school readiness, only half of the studies included all dimensions of school readiness. As such, research would benefit of a consensus on essential indicators of school readiness. Future studies should also continue to study school readiness with a person-centered approach to facilitate the screening of children most at risk of poor academic achievement and social adjustment and the need for early intervention.

### Supplementary Information

**Additional file 1**. Supplementary table: Table S1. Detailed description of the profiles of school readiness

## Data Availability

Not applicable.

## Abbreviations

B: Low-balanced
SE-C: Socioemotional difficulty
C + SE: High cognitive and low socioemotional skills
UC: Unbalanced cognition
C-SE: Cognitive difficulty
M: Moderate-Balanced
C + SE: Cognitive strength
TA: Tend to average
SE + C: High socioemotional and low cognitive skills
SE + C: Socioemotional strength
B +: High-balanced
T-: Tend to difficulties
SE↕: Average cognitive with mixed socioemotional skills
T +: Tend to strength
U: Unbalanced
GPA: Grade point average
