# Supplementary material for: School readiness among vulnerable children: a systematic review of studies using a person-centered approach
Source: Psicol Reflex Crit. 2024 Apr 17;37:16. doi: 10.1186/s41155-024-00298-y (PMC11024069; doi:10.1186/s41155-024-00298-y)
Supplement: Supplementary file 1 — Additional file 1. Supplementary table: Table S1. Detailed description of the profiles of school readiness [file 41155_2024_298_MOESM1_ESM.docx]

**Supplementary materials**

Table S1. Detailed description of the profiles of school readiness

| Authors | Proportion of children in each profile (%) | **Level in each domain of school readiness** | | | | |
| --- | --- | --- | --- | --- | --- | --- |
|  |  | Language skills | Cognitive | | Socio-  emotional | Motor/ physical |
|  |  |  | Pre-academic skills | Others |  |  |
| Tavassolie et al., 2022 | n/a | + | + |  | + | + |
|  | n/a | + | + |  | - | + |
|  | n/a | + | + |  | + | + |
|  | n/a | - | - |  | - | - |
|  | n/a | - | - |  | - | - |
|  | n/a | - | - |  | - | - |
| Fitzpatrick, 2017 | 57 | + | + | + | + |  |
|  | 34 | - | x | - | x |  |
|  | 9 | - | - | - | - |  |
| Christensen et al., 2020 | 70 | + | + | + | + | + |
|  | 16 | x | x | x | x | x |
|  | 7 | x | x | x | - | x |
|  | 7 | - | - | - | - | - |
| Hair et al., 2006 | 30 | + | + | + | + | + |
|  | 34 | - | - | - | + | + |
|  | 13 | - | - | - | - | - |
|  | 23 | - | - | - | x | - |
| Quirk et al., 2013 | 18 | + | + | + | + | + |
|  | 17 | - | - | - | + | x |
|  | 15 | x | x | x | x | x |
|  | 27 | - | - | - | - | x |
|  | 23 | - | - | - | - | - |
| Konold & Pianta, 2005 | 10 |  |  | - | x |  |
|  | 7 |  |  | - | x |  |
|  | 20 |  |  | x | x |  |
|  | 17 |  |  | x | - |  |
|  | 24 |  |  | x | + |  |
|  | 22 |  |  | + | - |  |
| McWayne et al., 2012a | 28 |  | x/x | x | +/x |  |
|  | 17 |  | +/- | - | - |  |
|  | 15 |  | x/x | x | -/x |  |
|  | 21 |  | x/x | + | + |  |
|  | 19 |  | +/+ | + | x |  |
| McWayne et al., 2012b | 31 |  | + | + | + |  |
|  | 38 |  | x | x | x |  |
|  | 31 |  | - | - | -/x |  |

low level (-); high level (+); average level (x); n/a= not available
